# Photoacoustic/ultrasound dual-modality imaging for marker clip localization in neoadjuvant chemotherapy of breast cancer

**DOI:** 10.1117/1.JBO.29.S1.S11525

**Published:** 2024-02-28

**Authors:** Handi Deng, Yizhou Bai, Jiaxuan Xiang, Zhaoyue Li, Peiliang Zhao, Yawen Shi, Wubing Fu, Yuwen Chen, Minggang Fu, Cheng Ma, Bin Luo

**Affiliations:** a Tsinghua University, Beijing National Research Center for Information Science and Technology, Department of Electronic Engineering, Beijing, China; b Tsinghua University, Institute for Precision Healthcare, Beijing, China; c Tsinghua University, Institute for Intelligent Healthcare, Beijing, China; d Beijing Tsinghua Changgung Hospital, Tsinghua University, School of Clinical Medicine, Beijing, China; e TsingPAI Technology Co., Ltd., Beijing, China; f Zhuhai Hospital Affiliated with Jinan University, Jinan University, Department of Thyroid and Galactophore Surgery, Zhuhai, China

**Keywords:** PA/US dual-modality imaging, marker clip, neoadjuvant chemotherapy, tumor localization

## Abstract

**Significance:**

To ensure precise tumor localization and subsequent pathological examination, a metal marker clip (MC) is placed within the tumor or lymph node prior to neoadjuvant chemotherapy for breast cancer. However, as tumors decrease in size following treatment, detecting the MC using ultrasound imaging becomes challenging in some patients. Consequently, a mammogram is often required to pinpoint the MC, resulting in additional radiation exposure, time expenditure, and increased costs. Dual-modality imaging, combining photoacoustic (PA) and ultrasound (US), offers a promising solution to this issue.

**Aim:**

Our objective is to localize the MC without radiation exposure using PA/US dual-modality imaging.

**Approach:**

A PA/US dual-modality imaging system was developed. Utilizing this system, both phantom and clinical experiments were conducted to demonstrate that PA/US dual-modality imaging can effectively localize the MC.

**Results:**

The PA/US dual-modality imaging can identify and localize the MC. In clinical trials encompassing four patients and five MCs, the recognition rate was ∼80%. Three experiments to verify the accuracy of marker position recognition were successful.

**Conclusions:**

We effectively localized the MC in real time using PA/US dual-modality imaging. Unlike other techniques, the new method enables surgeons to pinpoint nodules both preoperatively and intraoperatively. In addition, it boasts non-radioactivity and is comparatively cost-effective.

## 1 Introduction

The prevalence of breast cancer among women is steadily increasing, and neoadjuvant chemotherapy (NACT) remains a standard treatment.^1^^–^^3^ Compared with conventional methods, NACT has several advantages. First, it inhibits the metastasis of tumors by eradicating metastatic cells, thereby decreasing tumor severity and elevating breast conservation rates. Second, it offers insights into treatment efficacy. Should tumor growth be detected during this phase, the course of treatment (chemotherapy or surgical) might be modified. Third, reactions to NACT can function as an indicative measure for both disease-free survival and overall longevity outcomes.^4^^,^^5^ Depending on post-treatment pathological findings, adjustments in subsequent chemotherapy intensity, either an increase or decrease, can be determined. Once a complete tumor response is achieved through NACT, radiological and pathological examinations of the former tumor region post-treatment are necessary. To accurately pinpoint the location of the tumor, marker clips (MCs) are commonly used, which are implanted through either ultrasound (US) or mammographic imaging.^6^^,^^7^ However, US often falters in identifying the MC following NACT, especially in cases of total clinical response, prompting the need for mammography and incurring added radiation exposure.^8^^,^^9^

Photoacoustic (PA) imaging is an emerging technology which combines light and ultrasound for non-radioactive, highly sensitive functional and molecular imaging.^10^ At present, PA imaging has been applied to evaluate the effectiveness of NACT^11^^,^^12^ and to pinpoint seed implants in brachytherapy or metallic nanoparticles in soft tissues.^13^^–^^16^ Compared with US, PA imaging is superior in visualizing tiny metal implants. Yet, no studies have explored the feasibility of identifying MCs within breast tissue using PA imaging.

In this paper, we present MC imaging results in both phantom and clinical studies using a handheld PA/US dual-modality imaging system. We characterized the signal intensities and imaging features of MCs in agarose phantoms. Another imaging experiment involved an animal tissue phantom, and preliminary findings demonstrated that PA imaging exhibited superior specificity in distinguishing MCs from calcification and fascia compared to US imaging. However, US imaging proved more effective in differentiating small blood vessels that could be easily confused with marker signals under PA imaging. Therefore, PA/US dual-modality imaging has the potential to distinguish markers from breast tissue with improved specificity. Subsequently, we performed experiments on four volunteers to demonstrate the potential of PA/US imaging for accurate localization of the MCs in clinical settings. Finally, quantitative analysis was carried out on three patients to verify the accuracy of our proposed method.

## 2 Materials and Methods

### 2.1 Imaging System

**Fig. 1:**
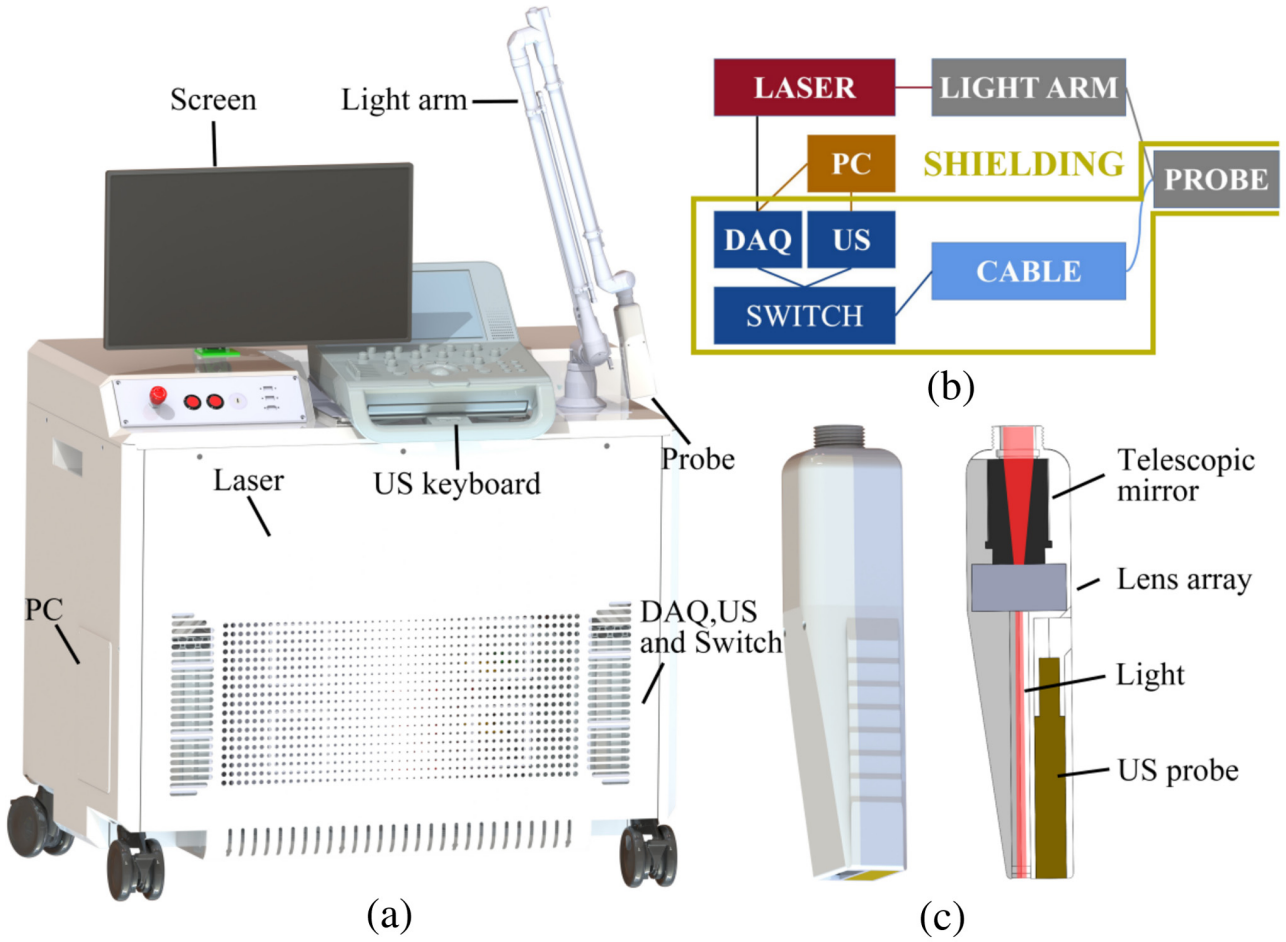
PA/US dual-modality imaging system. (a) 3D plot of the imaging system, (b) schematic diagram of key components, and (c) design of the PA/US probe. DAQ, data acquisition system; PC, personal computer; US, ultrasound.

Figures 1(a)–1(c) illustrate the PA/US system, a schematic detailing the system’s components, and the design of the dual-modal probe. Our system integrates a custom-built PA imaging system with a commercial US system (M_FE_A0, Stork Healthcare, China). A signal switch enables signal stitching between these two systems via a specially designed PA/US imaging probe, which features 128 elements, a center frequency of 5 MHz, and a −6  dB (US) bandwidth of 70%.

For the imaging probe, we employed a piezoelectric array with a polystyrene lens for elevational focusing at 2 cm. This approach offers the benefits of reduced sound attenuation and compatibility with optical coatings to enhance light reflection. For PA signal excitation, we used a laser (Nd: YAG; 1064-nm output, MQ/E, Beamtech Optronics, China) pulsing at 10 Hz with a pulse-width of 10 ns. The cost of a 1064 nm laser, capable of generating high-energy nanosecond pulses, can be as low as one to 2000 US dollars. This affordability has led to the widespread utilization of PA imaging systems based on 1064 nm lasers.^17^^,^^18^ Consequently, we employed a 1064 nm laser in our study. Our system’s design also permits multispectral PA imaging between 680 and 950 nm by replacing the laser with an optical parametric oscillator. The light beam was delivered via an articulated light arm, providing higher transmission efficiency than conventional fiber bundles. Figure 1(c) showcases the design of the imaging probe, which employed unilateral illumination. The laser beam from the articulated arm was first slightly focused through a series of lenses, and then reshaped and homogenized by a lens array. Finally, the laser beam formed a uniform 40  mm×10  mm rectangular pattern on the skin surface with an intensity of $∼50\;\mathrm{mJ}/{\mathrm{cm}}^{2}$, well below the safety threshold of $100\;\mathrm{mJ}/{\mathrm{cm}}^{2}$ at 1064 nm. The excited PA signals were captured using the MarsonicsDAQ128 system (Tianjin Langyuan Technology Co., LTD., China). Shielding was carefully applied to suppress electromagnetic interference. The shielding can mitigate the impact of pulsed radiofrequency radiation on PA imaging. The RF interference serves as a common-mode noise shared among all acquisition channels. In the case of linear array imaging probes, this common-mode noise presents itself as sharp horizontal lines that randomly appear in the PA image, impeding target identification. Therefore, shielding becomes imperative for enhancing target recognition efficiency. The received PA signals underwent high-pass filtering to eliminate low-frequency noise before image reconstruction using a delay-and-sum (DAS) algorithm. The features generated by bipolar signals are more conducive to the recognition of point-like targets, therefore we did not apply non-negative constraints or envelop detection to further convert the images to unipolar. Data processing accelerated by CUDA allowed real-time image display. We employ independent threads to reconstruct each pixel separately, significantly accelerating the reconstruction speed in the kernel function and the code is available in a GitHub repository.^19^ Our system was designed to simultaneously capture the dual-modality data, allowing for the co-display of PA/US images at a frame rate of 10 Hz. An interactive user interface offered control over reconstruction sound speed, imaging depth, and gain. All acquired data were saved for subsequent processing.

### 2.2 Marker Clip

The MC used in this study was the UltraClip™ Breast Tissue Marker (Catalogue No. 864017D, LOT No. HUEQ0525). This titanium alloy marker, with a size of 1 mm (diameter) × 3 mm (length), offers several notable features, including: (a) a rigid needle (with a needle size of 17 G × 10 cm) for independent placement, (b) suitability for marking during NACT, and (c) a design preventing migration.

### 2.3 Three-Dimensional Imaging of Marker Clip

To evaluate the PA signal intensity and imaging quality of the MC, we used a half-ring PA imaging system, comprising a 178 deg array (55 mm radius, 128 elements, 50 mm focal-length) and a fiber bundle. The fiber bundle incorporates 700 multi-mode fibers with a numerical aperture of 0.22. The output aperture size is 30  mm×1  mm. For stabilization, we employed a 3D-printed holder. The imaging chamber is soaked in water and the illumination plane intersects with the imaging plane at the sample position. Translation and rotation of the ultrasound array are achieved using a servo and a linear motor, respectively. This approach enables multi-angle joint reconstruction, resulting in a PA imaging system that is able to reconstruct the marker in three dimensions. For better display effects, in the maximum projection view, we set negative values to zero. The results of 3D imaging are talked about in Sec. 4.

### 2.4 Characterization of PA Signal Intensity of Marker Clip

We initially calibrated the signal intensity of the titanium wire (TA1) which is fabricated using the same material as the marker^20^ through phantom experiments to confirm that the MC was indeed a suitable target for PA imaging. The titanium wire and blood were securely held within a polytetrafluoroethylene (PTFE) tube with an inner diameter of 0.5 mm. Subsequently, we measured the PA signal intensity of the object at a wavelength of 1064 nm using a bright-field illuminated half-ring array imaging probe which is described in Sec. 2.3, with the imaging plane perpendicular to the PTFE tube. Initially, we inserted a titanium wire into the PTFE tube and filled it with water for acoustic coupling. Once we completed imaging of the titanium wire, it was retrieved, and the remaining water was drained from inside. Following this step, bovine blood was injected into the PTFE tube and imaged accordingly. The bovine blood was believed to be highly oxygenated.^21^ Throughout this process, the tube was kept stationary to prevent any influence on signal intensity caused by motion.

### 2.5 Phantom Experiment: Locating Marker Clip by PA Imaging

We performed PA imaging on MCs within phantoms to assess PA imaging’s ability to visualize the marker. We prepared the phantom by combining 3% agarose (G-10, Gene Company Ltd., Hong Kong) and 1% Intralipid (C14-24, 20%, Sichuan Kelun Pharmaceutical Co., Ltd., China) in water, yielding a $\mu_{\mathrm{eff}}$ of $∼0.4\;{\mathrm{cm}}^{-1}$. Initially, the marker was embedded in the agar phantom, and we performed 3D PA imaging by the system described in Sec. 2.3. Using the system detailed in Sec. 2.1, we further explored the marker’s imaging characteristics at various depths under limited-view conditions within the phantom. Specifically, MCs were introduced into two layers of agar blocks, which were coated with an ultrasonic coupling agent. By employing agar blocks of varying thicknesses, MCs were buried at depths of 5, 15, 25, and 35 mm, and we captured images along the long and short axes of the MCs. Following the agarose phantom study, we inserted MCs into a piece of pork and a piece of beef for PA/US dual-modality imaging. With the guidance of PA/US dual-modality imaging, we pinpointed the marker’s position using a puncture needle and affirmed the imaging accuracy through dissection.

### 2.6 Clinical Trial

Our prospective study received approval from the Medical Ethics Committee of Beijing Tsinghua Changgung Hospital and adhered to the guidelines of the Health Insurance Portability and Accountability Act (ethics approval number: 21181-0-01). We obtained written informed consent from all participants. Between February 2023 and July 2023, four subjects from Beijing Tsinghua Changgung Hospital took part in our study. We performed PA/US dual-modality imaging on these patients after implanting MCs in their breasts during the final surgical procedure. We employed the DAS algorithm incorporating angular weights for image reconstruction. During the dual-modality imaging process, the target area was first scanned along the length of the human body, followed by a horizontal scan. This procedure was video recorded, and snapshots of suspicious regions were taken.

The entry criteria for the clinical trial include: (1) Patients diagnosed with invasive breast cancer, as confirmed by core needle biopsy and immunohistochemical pathological examination. (2) Patients who had undergone preoperative NACT, either NACT alone or combined with targeted therapy, with the decision made by discussions with the multidisciplinary team. (3) Patients who consented to the placement of a MC (i.e., breast marker) into the breast lesion and any metastatic lymph nodes identified through biopsy either before or during NACT. (4) Patients who agreed to receive surgical operation for breast and axillary lesions post-NACT. The planned area of dissection must encompass, at a minimum, the lesions indicated by the marker, and the operation should incorporate the steps to locate and identify the marker. The exclusion criteria for the clinical trial include: (1) Patients for whom endocrine therapy was part of the NACT. (2) Patients who wish not or was not suitable for taking surgical operations after NACT. (3) Patients who declined timely mammography and breast-enhanced magnetic resonance imaging (MRI) examinations. (4) Patients with distant metastasis identified at the time of diagnosis.

The diagnosis and treatment process of the subjects was as follows. (1) US imaging was conducted initially (baseline) and then every 3 weeks during NACT. Mammography and MRI examinations were scheduled every 6 weeks to monitor the therapeutic response of the target lesions. The therapeutic outcomes were assessed based on the RECIST 1.1 criteria.^22^ (2) According to the patient’s wishes, markers were placed in the breast lesions and metastatic lymph nodes (if any) confirmed by biopsy before or 3–9 weeks after the initiation of NACT. (3) After the placement of the MC, the PA/US dual-modality imaging system was employed. Every 3 to 6 weeks, PA/US dual-modality imaging was carried out to capture the lesion where the marker was placed. The primary aim was to pinpoint the marker and evaluate the surrounding lesions.

To ensure the precise localization and extraction of the marker, we aim to confirm that images acquired from the PA/US imaging system accurately represent the marker’s location. Before surgery, we adhered to the following protocols for the imaging experiment: (1) Prior to the patient’s transition to the operating room, the marker is re-imaged and identified using the PA/US system. (2) If the marker is successfully identified without any special spatial relationship to tumors or lymph nodes, such as marker at the edge of the tumor or lymph, 0.02 to 0.05 ml of methylene blue (MB) is injected at the marker’s site, guided in real-time by the system’s image, after which the patient proceeds to surgery. (3) If the marker is not discernible through the PA/US system but a residual tumor or lymph node is visible under ultrasound, 0.02 to 0.05 ml of MB is injected to the residual tumor tissue, followed by the surgery. (4) In cases where the marker is indistinguishable via the PA/US system and no residual tumor or lymph node lesions are detected under ultrasound, patients scheduled for breast conserving surgery first undergo a mammography. Using mammography imaging, a mammography guided positioning wire is then placed near the marker, followed by the surgery. (5) Tissues excised during surgery are taken X-rayed, to confirm the position of markers. (6) Observing the blue stain on tissues surrounding the marker provides insight into the PA/US system’s reliability in pre-operative marker identification.

## 3 Results

Utilizing the method described in Sec. 2.2, we averaged and reconstructed the signals of the titanium wire and bovine blood 100 times. The normalized signal intensities for the titanium wire and blood were 1 and 0.91, respectively. Consequently, the titanium wire’s signal intensity was ∼1.09 times greater than that of the blood. Given that the titanium wire and blood have signal intensities of the same order of magnitude, the titanium wire is deemed suitable for PA imaging.

### 3.1 Phantom Imaging Results

**Fig. 2:**
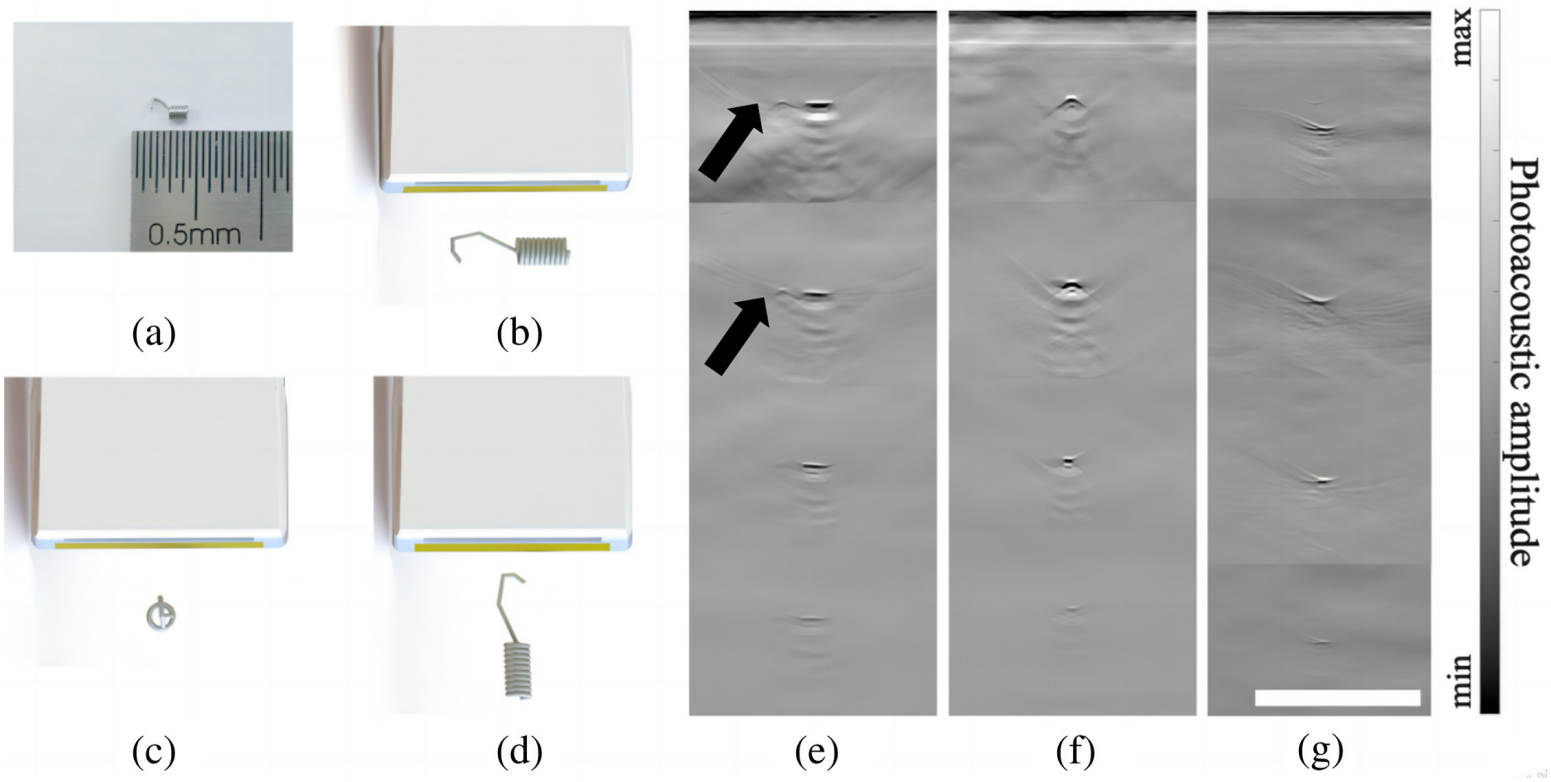
Photo and PA imaging results of markers. (a) Photographic depiction of the marker. (b)–(d) Three distinct marker orientations: (b) markers located within the imaging plane and aligned parallel to the array; (c) markers oriented perpendicular to the imaging plane; and (d) markers positioned within the imaging plane and perpendicular to the array. (e) PA imaging results of markers at depths of 5, 15, 25, and 35 mm, oriented as depicted in (b). (f) PA imaging results of markers at depths of 5, 15, 25, and 35 mm, oriented as illustrated in (c). (g) PA imaging results of markers at depths of about 5, 15, 25, and 35 mm, oriented as illustrated in (d). The scale bar in (g) is 10 mm. Black arrows point to the hook end of the marker.

Figure 2(a) displays a photograph of the MC. For the convenience of discussion, three representative marker orientations are illustrated in Figs. 2(b)–2(d). For better visualization, the markers’ sizes have been magnified eightfold. Figures 2(e)–2(g) show the PA images of markers at various depths, from 5 to 35 mm at a 10 mm interval which oriented as shown in Figs. 2(b)–2(d). The imaging results corresponding to the marker orientation in (b) exhibit features regardless of whether the hook is pointing to the left or to the right. Similarly, it makes no difference whether the hook points outward or inward in (c), or upward or downward in (d). Therefore, in the experiment, only one direction was chosen for each case. Note that in these images, both white and black represent strong PA pressure. In the colormap, black is labeled as “min,” indicating the highest negative PA pressure. According to Fig. 2(e), PA imaging was able to recover the marker’s shape, with signal strength decreasing at greater depths. Notably, at shallower depths, the hooked-shaped wire of the marker was discernible, as highlighted by the black arrows. Figure 2(f) further demonstrates that the marker’s coil was partially visible. However, as depth increased, such an image feature shrank into a point as the signal weakened, partially due to the limited-view effect. Figure 2(g) shows MCs, which, oriented as shown in Fig. 2(d), can also be identified but are easily confused with point features, such as blood vessels. These preliminary phantom tests validate the efficacy of PA imaging in capturing the markers’ features, suggesting its potential utility in tumor localization during NACT.

**Fig. 3:**
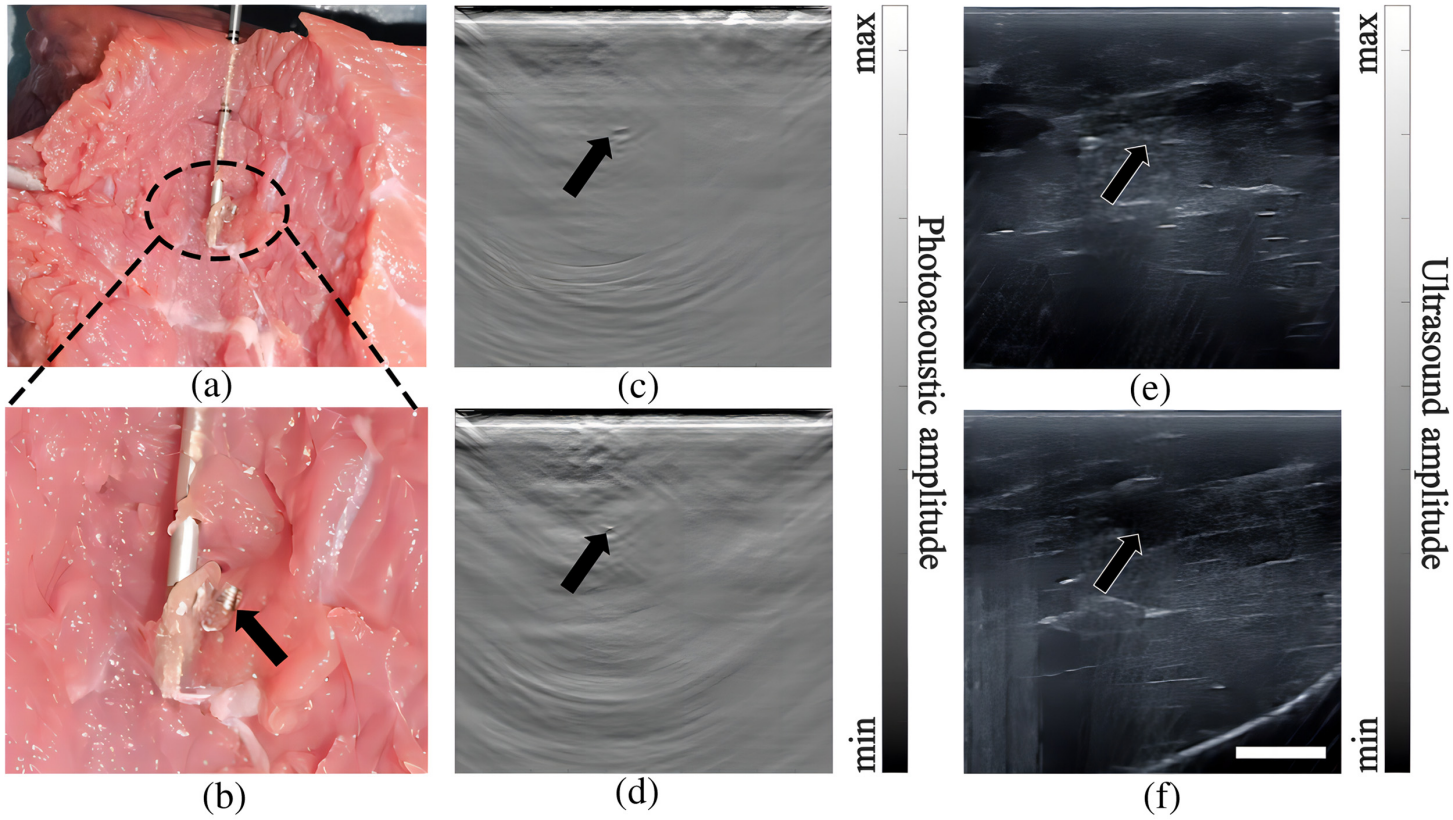
Photography and PA/US dual-modality imaging results of the marker localization in meat phantoms. (a), (b) Photos showing verification of marker location identified by PA/US dual-modality imaging. (c), (e) PA/US images of a marker oriented in the imaging plane and parallel with the linear array. (d), (f) PA/US images of a marker perpendicular to the imaging plane. Black arrows indicate the marker positions. The scale bar is 10 mm.

In the imaging experiments involving the beef and pork phantoms, MCs were placed at depths between 10 and 20 mm, and PA imaging was also proven to be effective in locating the markers. Figure 3 shows the results of the beef phantom experiment, with the black arrow pointing to the location of the marker. Figures 3(a) and 3(b) depict a photograph of the beef phantom, along with a zoomed-in view, illustrating the successful extraction of the marker. The needle in Fig. 3(a) was not present during the imaging process. After locating the MC, the needle was inserted into the MC position, guided by PA/US imaging. As confirmed by the photos depicted in Figs. 3(a) and 3(b), the needle was accurately positioned at the MC’s location. Figures 3(c) and 3(e) are the results of PA and US imaging when the imaging plane was perpendicular to the long axis of the marker. It can be seen that both modalities could image the marker. Since there were no large blood vessels in the beef phantom, the specificity of PA imaging was very high (there were no other strong signals except the marker). However, due to the abundant fascia in the muscle, there were many features that looked alike the marker in the US image, giving rise to a low specificity. Figures 3(d) and 3(f) show the results when the marker was aligned with the imaging plane. At this particular marker orientation, due to the reduction of effective ultrasound reflection, the marker was almost invisible in US image yet clearly visible in the PA image. Through these experiments, we have demonstrated that the integration of PA imaging with US imaging significantly enhances the capacity for MC detection. This underscores the importance of incorporating PA imaging for tumor localization during NACT.

### 3.2 Clinical Imaging Results and Statistics

**Fig. 4:**
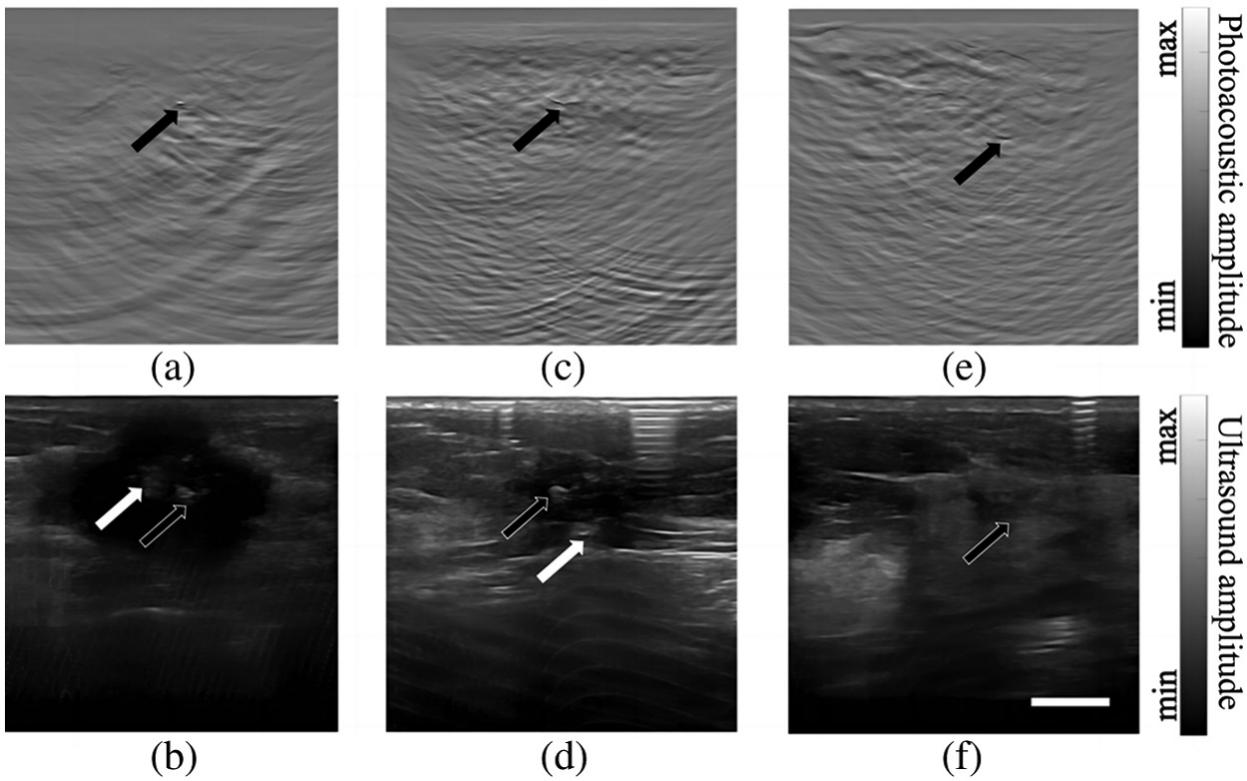
PA/US dual-modality imaging results of the breast positions of patients 1, 2, and 4. (a) and (b) PA and US images of patient 1, respectively; (c) and (d) PA and US images of patient 2, respectively; (e) and (f) PA and US images of patient 4, respectively. Black arrows represent true markers and white arrows point to fake signals that can be confused with the markers using US alone. The scale bar is 10 mm.

In this clinical study, we performed PA/US dual-modality imaging in four patients at different stages of treatment. The clinical information of the patients is shown in Table 1. Because medical iodophor disinfectant generates strong PA signals, the data obtained using iodophor as the coupling agent were excluded (lymph node region of patient 1). Therefore, the data of four breast markers (patient 1 to 4) and lymph node markers (patient 3) were used for clinical data analysis. With the exception of the marker in the breast region of patient 3, the remaining markers could be clearly distinguished. Figure 4 depict the PA/US images in the breast position of patients 1, 2, and 4. In the case of patients 1 and 2, the tumors did not completely disappear, and PA imaging identified the locations of the markers [indicated by black arrows in (a) and (c)]. The markers were also visible in US imaging [indicated by black arrows in (b) and (d)]. Nonetheless, differentiating the marker signal from neighboring calcification or fascia signals was difficult for US imaging, given the similarities in image intensity and morphology, as denoted by white arrows in (b) and (d). In the case of patient 4, although signals from superficial vessels could potentially introduce confusion, the PA signal originating from the marker remained visible and identifiable by a trained operator [Fig. 4(e), black arrow]. Significantly, in this instance, the marker’s signal was also discernible in the US image [Fig. 4(f), black arrow], reducing likelihood of confusion with blood signals in the PA image. In conclusion, these cases demonstrate that by using PA/US imaging, we can effectively pinpoint the markers.

**Table 1:** The clinical information of the patients.

No. of patient	Age of patient	Baseline TNM			Baseline HR, Her2 status		Number of markers		Time of placement	ypTNM and Miller–Payne grading of breast lesions after chemotherapy			
		T	N	M	HR	Her2	Breast	Axilla		ypT	ypN	ypM	Grade of MP
1	49	2	3	0	+	−	1	1	C1D15, week3	2	0	0	G2
2	49	2	0	0	+	+	1	0	C5D1, week9	Tis	0	0	G5
3	43	2	1	0	+	−	1	1	C4D3, week7	2	2	0	G3
4	51	2	0	0	−	+	1	0	C2D2, week4	0	2	0	G5

**Fig. 5:**
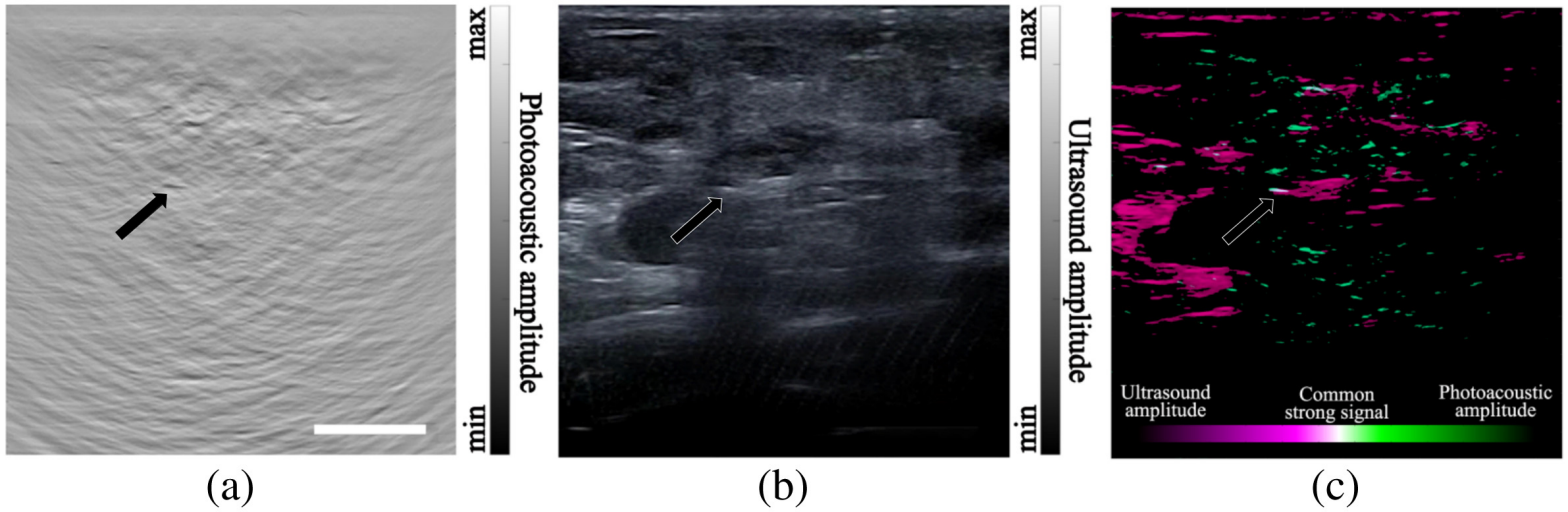
PA/US dual-modality imaging results of the lymph node location of patient 3. (a) PA image. (b) US image. (c) Co-plot of PA and US images using two different color schemes. Areas where the US and PA features overlap appear white. The arrows in these images point to the MC. The scale bar is 10 mm.

Figure 5 displays the imaging results for the lymph node region of patient 3. Similar to the earlier examples, the PA feature indicated by the black arrow in Fig. 5(a) can be identified as the MC. Its identity is further confirmed by the bright spot in the US image, as depicted by the arrow in Fig. 5(b). In Fig. 5(c), we rendered US features in purple and PA features in cyan. The regions where US and PA features overlap appeared white due to a blend of hues. The strongest white signal is indicated by the black arrow, demonstrating the location of the MC. Other pale white signals, visible in the ultrasound image, represent the boundaries of between different tissue types. Therefore, the identification of the marker’s location is more convenient through dual-modality imaging. Note that US imaging alone is not capable of affirmatively identify the marker due to similar signals from the surrounding tissues.

We summarize the imaging results in Table 2, categorizing the MCs into three groups: “clearly visible,” “barely visible,” and “invisible” for PA imaging alone, US imaging alone, and PA/US joint imaging. It is important to note that this classification is subjective and may depend on the operator’s experience. We anticipate that an operator with experience in US imaging, coupled with 1 h of training in PA imaging, would likely make similar judgments. During the imaging process, if a target could be confidently identified as an MC, we labeled it as “clearly visible.” If the target was discernible but the operator had reservations about its identity (such as when it was surrounded by similar signals), we labeled it as “barely visible.” Otherwise, if no signal from the MC was detected, we labeled the case as “invisible.” Notably, none of the MCs were categorized as “clearly visible” under US imaging alone. However, in the context of PA/US joint imaging during this clinical trial, we were able to confidently identify 4 out of 5 MCs.

**Table 2:** Marker detection results in PA imaging, US imaging, and PA/US joint imaging.

Region	Patient no.	PA imaging			US imaging			Dual-modality imaging		
		Clearly visible	Barely visible	Invisible	Clearly visible	Barely visible	Invisible	Clearly visible	Barely visible	Invisible
Breast	1	√	—	—	—	√	—	**√**	—	—
	2	√	—	—	—	√	—	**√**	—	—
	3	—	√	—	—	—	√	—	—	√
	4	—	√	—	—	√	—	**√**	—	—
Lymph node	3	—	√	—	—	√	—	**√**	—	—

### 3.3 Validation of Imaging Results

Due to surgical condition constraints, patient 2 could not participate in the validation of the imaging result, so the verification experiments were based on patients 1, 3, and 4. For patient 1, who received total mastectomy, we used fine needle puncture localization guided by PA/US imaging on surgically removed tissue and confirmed what we identified as markers by dissection.

**Fig. 6:**
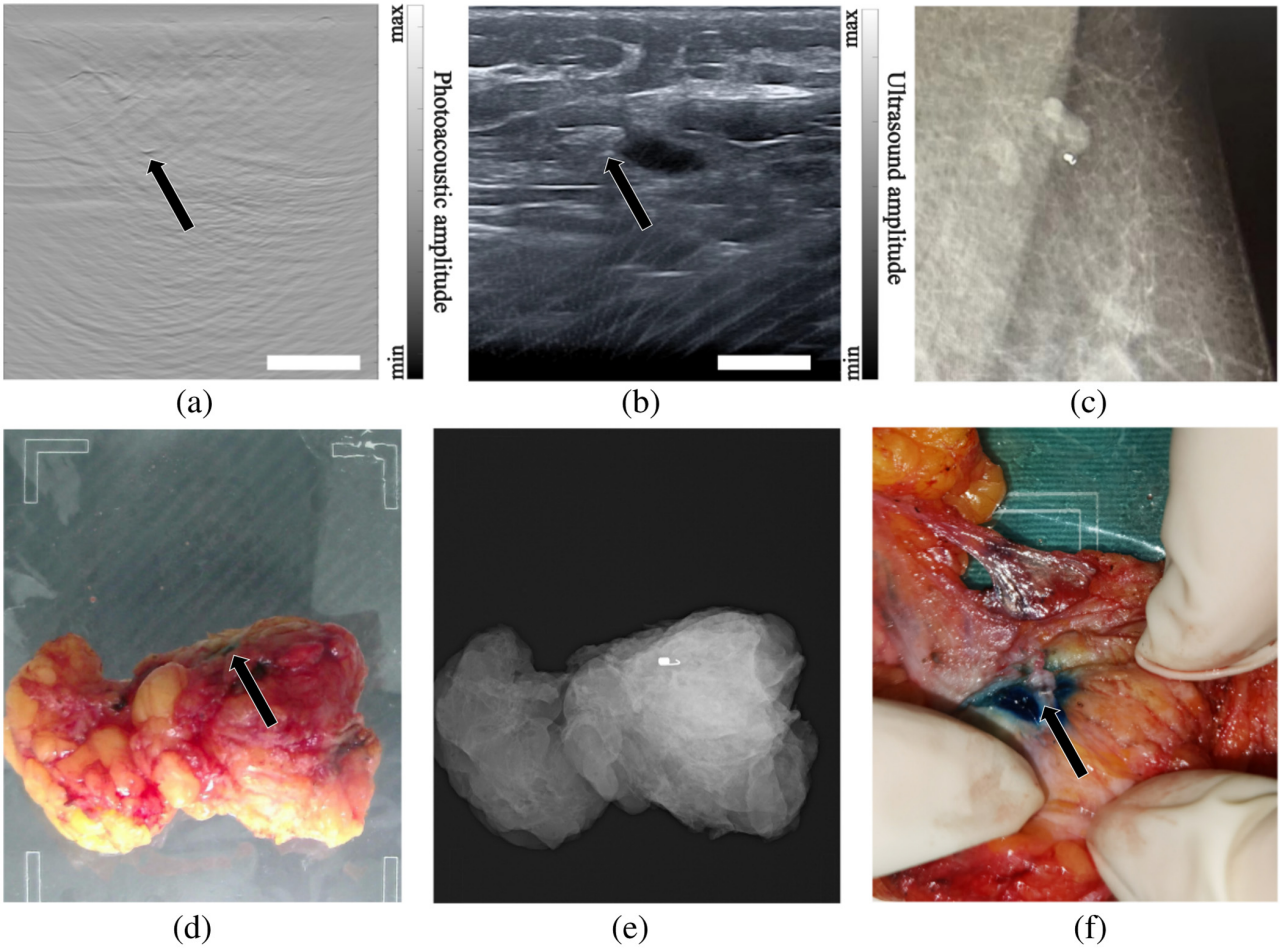
Validation of PA/US-based marker detection. (a) PA image of patient 3’s lymph node region; (b) US image of patient 3’s lymph node region; black arrows point to the MC. (c) Mammography shows that the marker was nearby a lymph node. (d) An *ex-vivo* tumor of patient 4, the black arrow indicates the location of the MB stain. (e) *Ex-vivo* mammography of the tumor. (f) Photograph of tissue dissection, the black arrow denotes the marker. The scale bar is 10 mm. (See Video 1, MP4, 2.19 MB [URL: https://doi.org/10.1117/1.JBO.29.S1.S11525.s1].)

In the case of patient 3, a distinctive image feature indicative of the MC was identified in proximity to the lymph node location on the PA images, as shown in Fig. 6(a). This discernible image feature was subsequently verified in the corresponding US images, as depicted in Fig. 6(b). The joint analysis of the PA/US images provided additional confirmation of its classification as an MC, clearly revealing its location at the periphery of a lymph node. This spatial relationship was further verified through mammography, confirming the marker’s proximity to the side of a lymph node, as shown in Fig. 6(c).

Patient 4 chose breast-conserving surgery, and we performed MB injection as detailed in Sec. 2.4 to validate the findings of PA/US imaging. The actual injection position was above the marker position, and the injection process was shown in Video 1. A photo of the surgically removed tumor is shown in Fig. 6(d), while the X-ray image of the *ex vivo* tumor is shown in Fig. 6(e). Since the two images were taken by the same equipment, their positions were well aligned. The *ex vivo* tumor was oriented with its superficial aspect facing upward to faithfully reproduce its original orientation. As shown in Figs. 6(d) and 6(f), the marker was situated directly beneath the blue-stained area, consistent with its position in the PA/US images. This confirms that the object we identified was indeed an MC. In conclusion, the above three validation experiments attest to our method’s effectiveness in identifying MCs *in vivo*.

### 3.4 Cause of Identification Failure

**Fig. 7:**
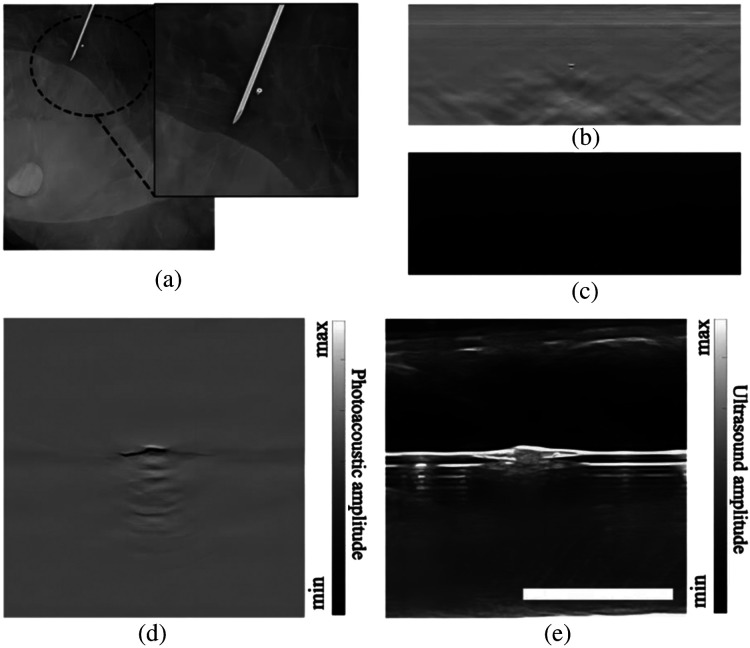
Analysis of marker detection failure in the breast region of patient 3. (a) Mammography image of *ex-vivo* breast tissue of patient 3. Zoomed-in view shows the marker (normal to the imaging plane) near a needle. (b) PA image of a marker placed vertically. (c) US image of the vertical marker. (d) PA image of the same marker when it was horizontal. (e) US image results of the horizontal marker. The scale bar is 10 mm.

The marker embedded in the breast of patient 3 could not be accurately identified. After undergoing a total resection, the excised specimen from patient 3 was subjected to post-operative mammography. The specimen was properly oriented to replicate the *in-vivo* breast position. As shown in Fig. 7(a), the marker appeared to be oriented vertically to the skin, due to the contraction of the breast tumor during NACT. To further analyze this special case, we performed a study where the original marker was vertically inserted into an agar phantom, followed by dual-modality imaging. The results, presented in Figs. 7(b) and 7(c), demonstrate that the marker’s feature in the PA image resembled a point, which can be easily mistaken for a small vessel perpendicular to the imaging plane. Concurrently, the US imaging failed to capture any signal from the marker. Subsequently, we flipped the marker by 90 deg, aligning its long axis with the transducer array, and the imaging results are shown in Figs. 7(d) and 7(e). It can be seen that in this new position, the MC was clearly visible in the PA/US images, excluding the possibility that this particular marker had a weakened signal. It is worth noting that PA/US dual-modality imaging does not impact the localization of MCs by traditional mammography. Therefore, for the minority of patients whose MCs are not found in PA/US imaging, they can use mammography to locate MCs as usual.

## 4 Discussion and Conclusions

We employed a handheld PA/US dual-modality imaging system to identify MCs in real-time with improved accuracy. PA/US dual-modality imaging has shown to be a non-radioactive and cost-effective potential tool enabling surgeons to pinpoint the marker both pre-surgery and intraoperatively. To more accurately quantify the benefits of PA/US dual-modality imaging, it is essential to undertake more in-depth research and accumulate a larger volume of clinical data. Furthermore, to further improve the MC detection rate, we aim to enhance the sensitivity of the ultrasonic transducer. Deep signals can be overwhelmed by reflection artifacts,^23^^,^^24^ and devising strategies to mitigate these artifacts is an effective way to enhance the performance. Collaborating with MC manufacturers to customize markers with isotropic signal emission can boost the efficacy of PA/US dual-modality imaging.

**Fig. 8:**
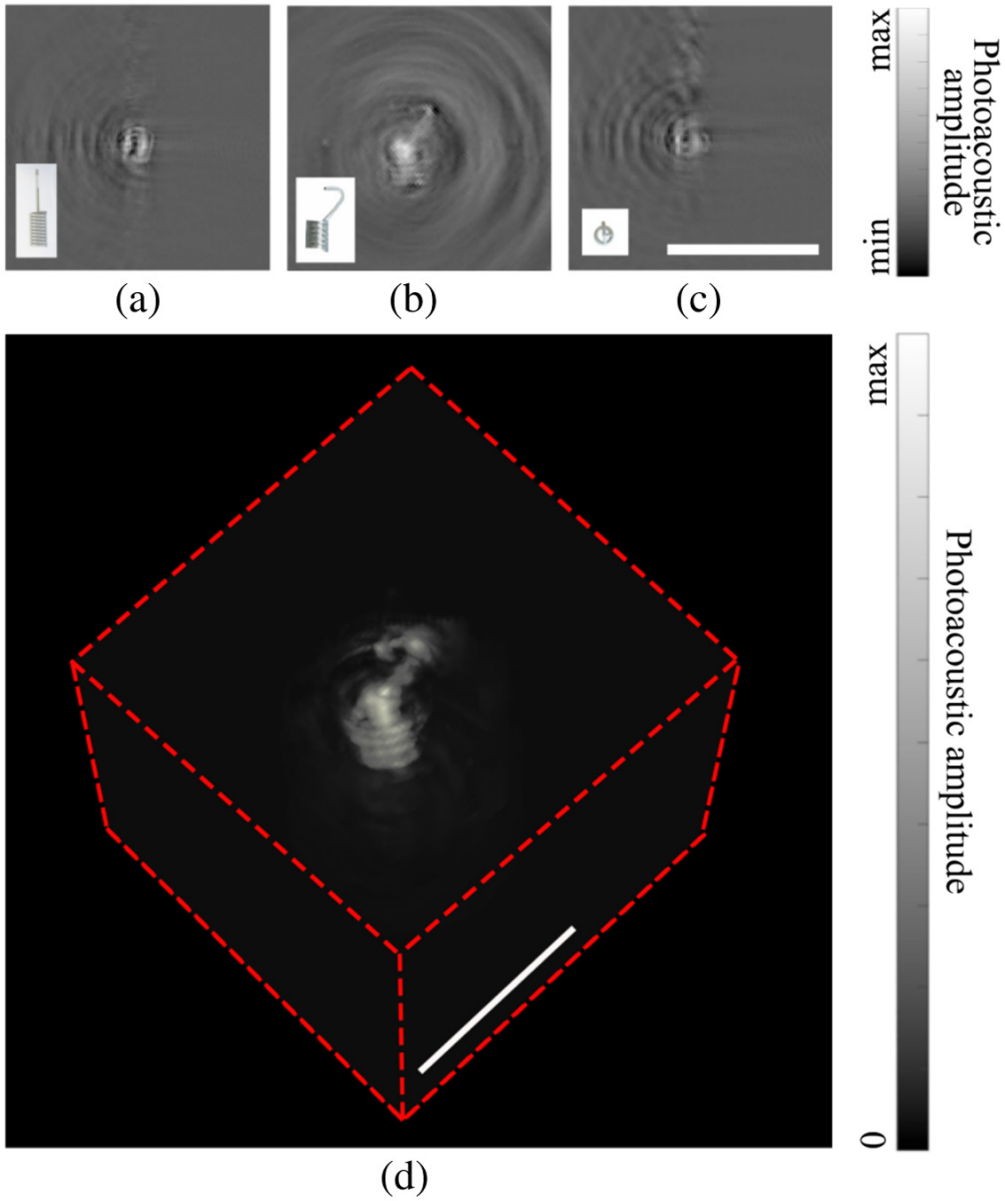
3D PA imaging results of a marker. (a)–(c) Cross-sectional displays of the 3D PA image in three orthogonal directions. (d) Maximum intensity projection of the 3D PA image in a volumetric rendering. All scale bars represent 5 mm.

A challenge in both PA and US imaging of MCs is the difficulty in distinguishing marker signals from intrinsic tissue signals, such as blood vessels. Deep learning for feature recognition can be used in conjunction with PA/US imaging for higher detection rate and speed.^8^ Handheld 3D PA imaging is also a promising solution for enhancing marker detectability. From Fig. 8, we conclude that 3D PA imaging effectively captures the overall morphological features of an MC. We note that 3D PA imaging^18^^,^^25^ is superior in directly distinguishing between MCs and blood vessels, a differentiation that can be challenging to achieve in two-dimensional imaging. Moreover, multi-spectral PA imaging^26^ might improve marker identification by identifying characteristic PA spectra from the marker. In summary, PA/US dual-modality imaging system is a promising tool for locating MCs.

## Supplementary Material



## Data Availability

A part of the reconstruction code is available at GitHub - https://github.com/Adi-Deng/photoacoustic-TsingPAI-Co.-LTD.-. The other code and data underlying the results presented in this paper are not publicly available at this time but may be obtained from the authors upon reasonable request.
